# AAV6-based ZEB2 delivery promotes cardiomyocyte dedifferentiation in adult human myocardium

**DOI:** 10.1093/cvr/cvaf190

**Published:** 2025-10-10

**Authors:** Rocco Caliandro, Azra Husetić, Merel L Ligtermoet, Jermo Hanemaaijer-van der Veer, Karel van Duijvenboden, Lorena Zentilin, Mara Clerkx, Mauro Giacca, Roelof-Jan Oostra, Maurice J B van den Hoff, Vincent M Christoffels, Monika M Gladka

**Affiliations:** Department of Medical Biology, Amsterdam Cardiovascular Sciences, Amsterdam University Medical Centers, University of Amsterdam, 1105 AZ Amsterdam, The Netherlands; Department of Medical Biology, Amsterdam Cardiovascular Sciences, Amsterdam University Medical Centers, University of Amsterdam, 1105 AZ Amsterdam, The Netherlands; Department of Cell and Chemical Biology, Leiden University Medical Center, Leiden, The Netherlands; Department of Medical Biology, Amsterdam Cardiovascular Sciences, Amsterdam University Medical Centers, University of Amsterdam, 1105 AZ Amsterdam, The Netherlands; Department of Obstetrics and Gynaecology, Amsterdam University Medical Centers, University of Amsterdam, Amsterdam, The Netherlands; Amsterdam Reproduction and Development Research Institute, Amsterdam, The Netherlands; Department of Medical Biology, Amsterdam Cardiovascular Sciences, Amsterdam University Medical Centers, University of Amsterdam, 1105 AZ Amsterdam, The Netherlands; AAV Vector Unit, International Centre for Genetic Engineering and Biotechnology (ICGEB), Trieste, Italy; Department of Medical Biology, Amsterdam Cardiovascular Sciences, Amsterdam University Medical Centers, University of Amsterdam, 1105 AZ Amsterdam, The Netherlands; School of Cardiovascular and Metabolic Medicine & Sciences and British Heart Foundation Centre of Research Excellence, King’s College London, London SE5 9NU, UK; MRC/BHF Centre of Research Excellence in Advanced Cardiac Therapies (REACT), King’s College London, London SE5 9NU, UK; Department of Medical Biology, Amsterdam Cardiovascular Sciences, Amsterdam University Medical Centers, University of Amsterdam, 1105 AZ Amsterdam, The Netherlands; Department of Medical Biology, Amsterdam Cardiovascular Sciences, Amsterdam University Medical Centers, University of Amsterdam, 1105 AZ Amsterdam, The Netherlands; Department of Medical Biology, Amsterdam Cardiovascular Sciences, Amsterdam University Medical Centers, University of Amsterdam, 1105 AZ Amsterdam, The Netherlands; Department of Medical Biology, Amsterdam Cardiovascular Sciences, Amsterdam University Medical Centers, University of Amsterdam, 1105 AZ Amsterdam, The Netherlands

**Time of primary review: 30 days**

Zinc Finger E-box Binding Homeobox 2 (ZEB2) is a key regulator of epithelial-to-mesenchymal transition (EMT), an essential process for tissue remodelling and wound healing.^1^ We previously showed that the Adeno-Associated Virus serotype 9 (AAV9)-mediated ZEB2 delivery *in vivo* facilitates the crosstalk between cardiomyocytes (CMs) and endothelial cells (ECs), enhancing ECs’ proliferation and migration and promoting infarct healing and cardiac repair.^2,3^ Similarly, ZEB2 delivery induces angiogenesis in *ex vivo* adult pig myocardial tissue slices.^4^

To gain insight into the cardioprotective effects of AAV-delivered ZEB2 in human cardiac tissue, we utilized an *ex vivo* model of living myocardial slices (LMSs). Human LMSs provide significant advantages for disease modelling and drug testing, as they preserve the tissue architecture of the adult heart, maintain intact cell-cell interactions across all cardiac cell types, and retain pathophysiological states resulting from comorbidities experienced by the donor during their lifetime. All procedures involving human participants complied with relevant ethical regulations and were approved by the institutional review board of Amsterdam University Medical Centres (2024.0643). All donors gave written informed consent and the study conformed to the principles of the Declaration of Helsinki. We isolated the heart of a 74-year-old female donor [affected by systolic dysfunction (HFrEF), atrial fibrillation and obesity] and used it to prepare 300 µm LMSs, cultured for a total of 4 days, using the air-liquid interface method (*Figure 1A*). For transgene delivery, we utilized CMV-driven AAV6 carriers (*Figure 1B*), known for their excellent gene transfer efficiency in LMSs (MOI 20000), as previously reported.^4,5^ Transduction efficiency was confirmed by visualization of AAV6-delivered GFP (*Figure 1C*). Consistent with previous findings,^3^ delivery of ZEB2 resulted in enhanced angiogenesis as shown by significantly increased VWF^+^ vessel areas compared with control (*Figure 1D* and *E*).

**Figure 1:**
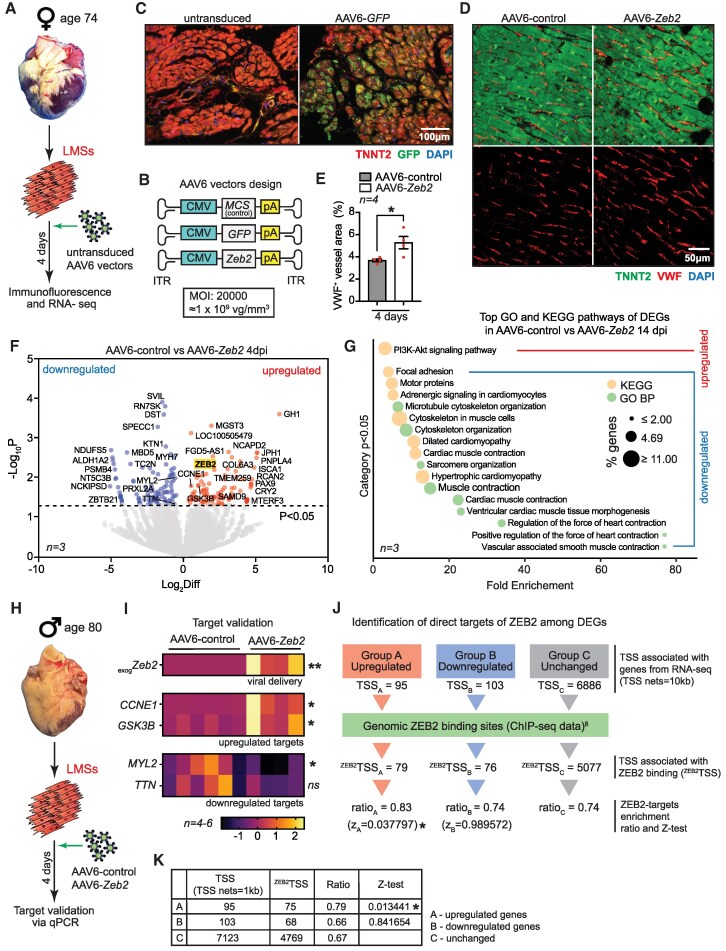
ZEB2 drives dedifferentiation in adult human cardiomyocytes. (*A*) Study design. (*B*) AAV6 vectors design. (*C*) Representative immunofluorescent images of LMSs untransduced and transduced with AAV6-*GFP*. LMSs were stained for TNNT2 (red), GFP (green) and DAPI (blue). (*D*) Immunofluorescence for VWF (red), TNNT2 (green) and DAPI (blue) in LMSs transduced with AAV6-control and AAV6-*Zeb2.* (*E*) Quantification of VWF^+^ (positive) vessels area in LMSs treated with AAV6-control and AAV6-*Zeb2* (*P < 0.05; Student’s *t*-test, LMSs *n = 4*), bars show the mean of replicates ± SEM. (*F*) Volcano plot of DEGs in AAV6-*Zeb2* compared with AAV6-control treated LMSs. In blue—down-regulated genes, in red—up-regulated genes. Overexpression of ZEB2 is marked in yellow. (*G*) Functional enrichment analysis of RNA-seq data generated using AAV6-control- or AAV6-Zeb2-transduced LMSs. Top significant terms from Gene Ontology and KEGG pathways are displayed (*P < 0.05*). (*H*) Study design. (*I*) Heatmap of the relative expression of *exogenous Zeb2* (*_exog_Zeb2*) and some of the DEGs identified with RNA-seq in AAV6-control- or AAV6-*Zeb2*-transduced LMSs. (*P < 0.05, **P < 0.01; Student’s *t-test*, LMSs *n = 4–6*), data are presented as *Z*-score of *HPRT1* normalized expression. (*J*) RNA-seq and Chip-seq integration analysis workflow (with TSS nets = 10 kb). Group (*A*): up-regulated genes in RNA-seq, Group (*B*): down-regulated genes in RNA-seq, Group (*C*): unchanged genes in RNA-seq. For group C cutoff of >1 for the baseline value reported by DESeq2 was used. (*P < 0.05; *Z*-test). (*K*) RNA-seq and Chip-seq integration analysis (with TSS nets = 1 kb). Group (*A*): up-regulated genes in RNA-seq, Group (*B*): down-regulated genes in RNA-seq, Group (*C*): unchanged genes in RNA-seq. For group C cutoff of >1 for the baseline value reported by DESeq2 was used. TSS, Transcription Start Site. (*P < 0.05; *Z*-test).

Total RNA was isolated from AAV6-treated LMSs and used for library prep (Ovation RNA-seq System V2, Tecan genomics, #7102-31) and subsequent RNA-seq (NovaSeq, Illumina). DESeq2 analysis (Galaxy Tool Version, 24.2.4dev0) was performed to identify Differentially Expressed Genes (DEGs) in AAV6-*Zeb2*-treated LMSs compared with control (*Figure 1F*).

Pathway analysis (DAVID, v2024q4) of RNA-seq data revealed activation of genes associated with the PI3K-Akt signalling pathway in AAV6-*Zeb2*-transduced LMSs (*Figure 1G*). The PI3K-Akt signalling pathway is essential for cardiomyocyte growth, survival, and cardioprotection.^6^ Down-regulated genes are involved in cardiac muscle contraction, cytoskeleton, microtubule and actin filament organization, processes that characterize adult CMs (*Figure 1G*). The decrease in these processes could represent the first step towards cardiomyocyte dedifferentiation, typically characterized by enhanced cellular plasticity and partial cell cycle re-entry, resembling an EMT-like regenerative state.^7^ In the adult mammalian hearts, cardiomyocyte dedifferentiation rarely leads to cell proliferation, but instead involves a regression from mature functions such as oxidative metabolism and contractile activity, toward a less specialized, developmentally immature state.^8^ This shift reduces energy demands and offers cardioprotective benefits, which may resemble an evolutionary remnant of ancestral regenerative mechanisms.

Target validation was performed by qPCR using LMSs derived from the heart of a second donor (80-year-old male with type-2 diabetes, hypertension and asthma/COPD) (*Figure 1H*). After confirming delivery of exogenous *Zeb2* (*_exog_Zeb2*), we tested some of the DEGs identified via RNA-seq, observing similar changes in gene expression (*Figure 1I*).

To test whether direct target genes of ZEB2 are enriched among the DEGs, we determined the frequency of ZEB2 binding at ±10 or ±1 kbp around the transcription start site (TSS) of genes the expression of which was up-regulated (Group A), down-regulated (Group B) or unchanged (Group C) (*Figure 1J*). Genomic ZEB2 binding sites were retrieved from an available ChIP-seq dataset.^9^ Reads were mapped to the hg19 human genome using BWA with default parameters. The replicates were merged, and BEDTools was used to partition the genome into 1000 bp bins with a 500 bp sliding window.^10^ For peak calling, bins with >500 tags were merged, generating 43 069 individual genomic sites associated with ZEB2. TSSs were derived from the hg19 genome (GRCh37.p13) via Biomart and overlapping TSSs were merged. TSS ±10 kbp or ±1 kbp of differentially expressed and control gene sets were analysed for overlap with ZEB2 ChIP-seq peaks using BEDTools.^10^ We found that TSSs of up-regulated genes (Group A) are most frequently associated with ZEB2 ChIP-seq peaks, regardless of whether a 10 kb (*Figure 1J*) or 1 kb window around the TSS was analysed (*Figure 1K*). This corroborates our earlier findings that ZEB2 functions primarily as a transcriptional activator rather than a repressor in the adult mammalian heart.^3^

In this study, consistent with our previous findings in mouse heart and pig-derived LMSs, we show that ZEB2 delivery increases the number of ECs in human-derived LMSs. We also found transcriptional evidence suggestive of cardiomyocyte dedifferentiation, a prerequisite for heart regeneration.^7^ Further testing in a larger sample size is required to corroborate these observations and assess at the functional level the extent to which human adult CMs dedifferentiate in an EMT-like manner. Additionally, the long-term effects of a ZEB2-based cardiac therapy in human-derived LMSs need to be addressed to fully explore the benefits and drawbacks of delivering ZEB2 to the human heart. Despite these limitations, this study pioneers the impact of AAV6-mediated *Zeb2* gene transfer to adult human myocardium, demonstrating the potential applicability of AAV-based *Zeb2* therapy to promote cardiac repair in human heart tissue.

## Data Availability

Data including transcriptome analyses can be obtained from the authors upon request.
